# Genome-Wide Analysis of *SAUR* Gene Family Identifies a Candidate Associated with Fruit Size in Loquat (*Eriobotrya japonica* Lindl.)

**DOI:** 10.3390/ijms232113271

**Published:** 2022-10-31

**Authors:** Ze Peng, Wenxiang Li, Xiaoqing Gan, Chongbin Zhao, Dev Paudel, Wenbing Su, Juan Lv, Shunquan Lin, Zongli Liu, Xianghui Yang

**Affiliations:** 1 State Key Laboratory for Conservation and Utilization of Subtropical Agro-Bioresources, South China Agricultural University, Guangzhou 510642, China; 2 Key Laboratory of Innovation and Utilization of Horticultural Crop Resources in South China, Ministry of Agriculture and Rural Affairs, College of Horticulture, South China Agricultural University, Guangzhou 510642, China; 3 Department of Environmental Horticulture, Gulf Coast Research and Education Center, IFAS, University of Florida, Wimauma, FL 33598, USA; 4 Fruit Research Institute, Fujian Academy of Agricultural Science, Fuzhou 350013, China; 5 College of Materials and Energy, South China Agricultural University, Guangzhou 510642, China

**Keywords:** auxin, cell expansion, fruit size, loquat, *SAUR*

## Abstract

Fruit size is an important fruit quality trait that influences the production and commodity values of loquats (*Eriobotrya japonica* Lindl.). The *Small Auxin Upregulated RNA* (*SAUR*) gene family has proven to play a vital role in the fruit development of many plant species. However, it has not been comprehensively studied in a genome-wide manner in loquats, and its role in regulating fruit size remains unknown. In this study, we identified 95 *EjSAUR* genes in the loquat genome. Tandem duplication and segmental duplication contributed to the expansion of this gene family in loquats. Phylogenetic analysis grouped the *SAURs* from *Arabidopsis*, rice, and loquat into nine clusters. By analyzing the transcriptome profiles in different tissues and at different fruit developmental stages and comparing two sister lines with contrasting fruit sizes, as well as by functional predictions, a candidate gene (*EjSAUR22*) highly expressed in expanding fruits was selected for further functional investigation. A combination of Indoleacetic acid (IAA) treatment and virus-induced gene silencing revealed that *EjSAUR22* was not only responsive to auxin, but also played a role in regulating cell size and fruit expansion. The findings from our study provide a solid foundation for understanding the molecular mechanisms controlling fruit size in loquats, and also provide potential targets for manipulation of fruit size to accelerate loquat breeding.

## 1. Introduction

Auxin plays a key role in regulating the growth and development of plants, which is achieved through activities such as controlling the plasma membrane H^+^-ATPase, or the regulation of plant genes [1,2]. The auxin-responsive genes in plants were classified into many gene families, including the *Auxin/Indoleacetic Acid* (*Aux/IAA*), *Gretchen Hagen 3* (*GH3*), *Small Auxin-Up RNA* (*SAUR*), *Auxin Response Factor* (*ARF*), and *Glutathione S-transferase* (*GST*) families [3,4,5,6]. Among the three early auxin-responsive gene families (*Aux/IAA*, *GH3*, and *SAUR*), the *SAUR* genes can quickly respond to auxin stimuli within minutes, indicating that their transcription is greatly affected by auxin [7].

The first *SAUR* gene was identified in soybeans in their elongating hypocotyls, and was found to be rapidly induced by auxin [8]. In the past two decades, the *SAUR* gene families have been analyzed in many plant species, such as *Arabidopsis* [4], rice [9], sorghum [10], maize [3], citrus [11], watermelon [12], and apple [13]. It has been reported that many members of the *SAURs* family were derived from tandem duplications and segmental duplications, resulting in functional redundancy in some *SAUR* paralogues [14]. Despite the availability of these studies, few studies reported the functional characterization of the *SAUR* genes.

Various functions of *SAURs* have been reported from studies in *Arabidopsis*. The overexpression of *AtSAUR32* contributes to a hookless phenotype in *Arabidopsis*, which can be rescued by exogenous auxin [15]. *AtSAUR63* plays a role in cell elongation, and its overexpression can lead to the elongation of several tissues, such as the hypocotyls, petals, and stamen filaments [16]. The overexpression of *AtSAUR36* and *AtSAUR49* promotes leaf senescence [17,18]. The *AtSAUR19* subfamily was reported to play a role in promoting cell expansion [19]. In addition, studies have shown that many *SAURs* participate in the synthesis and transport of auxin, and some members may play a positive role in fruit expansion [20,21].

Cultivated loquat (*Eriobotrya japonica* Lindl.) is a fruit tree crop in *Rosaceae*. Fruit quality traits greatly influence its production and commodity values, which makes them an important aspect of loquat research, for example, fruit weight/size [22,23,24]. Research has shown that cell number and cell size are two key factors determining the fruit size, while auxin is capable of promoting cell division and elongation [25]. Therefore, the levels and signal transduction of auxin are closely related to fruit size [26]. Despite the availability of several reference genomes for loquats [24,27,28], currently, no studies have been performed to analyze the *SAUR* gene family systematically and comprehensively in loquats. This has limited our understanding of the role of *SAURs* in regulating fruit weight/size in loquats. In this study, we performed a genome-wide analysis of the *SAUR* gene family in loquats and identified a candidate *SAUR* associated with cell expansion and fruit size. The findings from our study provide valuable information for fruit size breeding in loquats and lay a solid foundation for understanding the molecular mechanisms controlling fruit size in loquats.

## 2. Results

### 2.1. Identification and Annotation of the SAUR Family

Based on the reference genome of ‘Seventh Star’ [24], a total of 95 EjSAUR genes were identified from the loquat genome (Table 1). The EjSAURs were named EjSAUR1-EjSAUR95 according to their locations (chromosome one-seventeen, top to bottom). The predicted peptide lengths ranged from 90 to 226 amino acids, the predicted molecular mass ranged from 9.98 to 25.48 kDa, while the theoretical PI ranged from 4.92 to 10.14 (Table 1). The prediction of subcellular localization revealed that the majority of the EjSAURs are localized in the mitochondria (43) and nucleus (31) (Table 1).

### 2.2. Phylogenetic Relationships, Gene Structure, and Conserved Motifs

**Figure 1:**
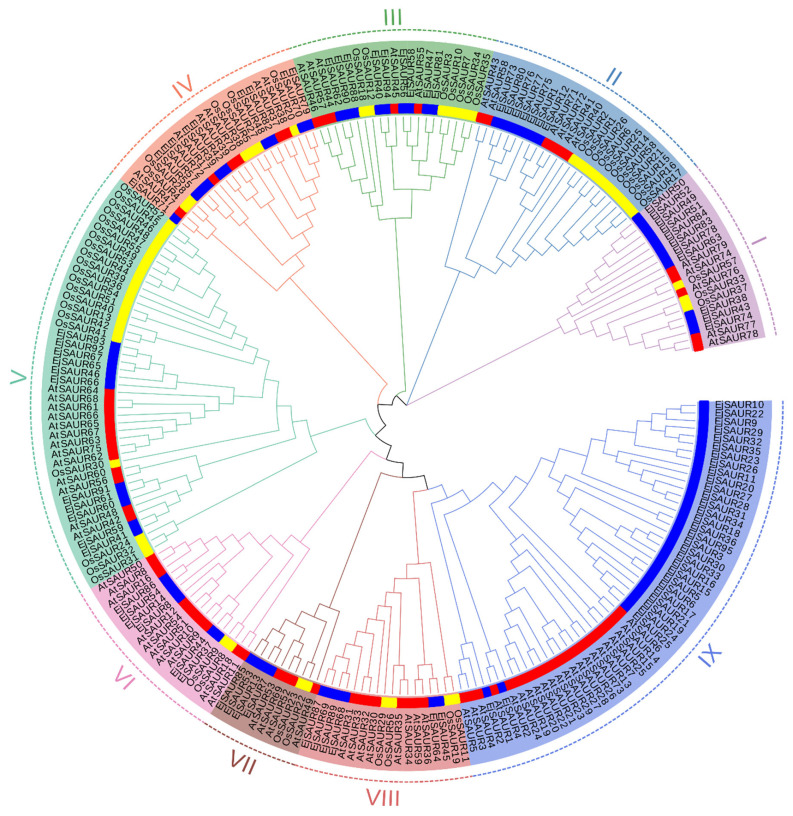
Unrooted neighbor-joining phylogenetic tree of *small auxin-up RNAs* (*SAURs*) in loquats, Arabidopsis, and rice. The phylogenetic tree was constructed using SAUR protein sequences from Arabidopsis (red), rice (yellow), and loquats (blue) using the neighbor-joining method in MEGA11. A total of 1000 bootstraps were used. The genes belonging to different clusters (I–IX) are highlighted in different colors.

To investigate the phylogenetic relationships among the *EjSAURs* as well as the *SAURs* from *Arabidopsis* and rice, a phylogenetic tree was constructed based on their protein sequences using the neighbor-joining method in MEGA11 (Figure 1 and Table S1). The *EjSAURs* together with other *SAURs* were assigned to nine different clusters. Clusters IX and V contained the largest numbers of *SAURs* (53 and 45). Cluster VII contained the smallest numbers of *SAURs*, including two *OsSAURs*, four *EjSAURs*, and four *AtSAURs*. The majority of the *EjSAURs* were assigned to Cluster IX, similar with *Arabidopsis*, while most of the *SAURs* from rice were assigned to Clusters II and V. Interestingly, Cluster IX only contained *EjSAURs* and *AtSAURs*, which suggested that loquats and *Arabidopsis* (both dicots) may have retained the most duplication events of the *SAUR* gene families during evolution.

**Figure 2:**
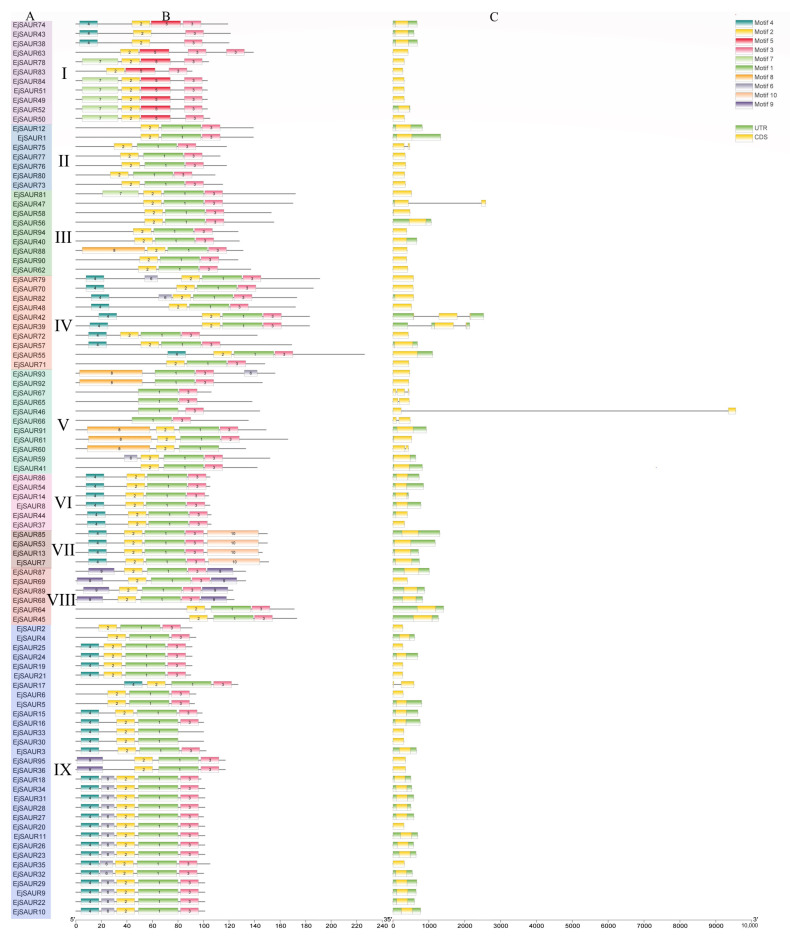
Conserved motifs and exon–intron structures of the predicted EjSAUR proteins. (**A**) EjSAURs of different clusters (I–IX). (**B**) Each motif is represented by a colored box. (**C**) Exon–intron structures of EjSAUR proteins. The exons and introns are represented by boxes and gray lines, respectively.

The exon–intron structures of *EjSAURs* are shown in Figure 2. Most members of the *SAUR* gene families contained no intron. A total of 85 *EjSAURs* did not contain an intron, which is consistent with the cases reported in other plant species. Among the remaining 10 *EjSAURs*, seven contained one intron, while the other three contained two introns. Interestingly, most members of Clusters VI, VII, VIII, and IX contained UTRs. Most members of Clusters VI and VII contained long 5′ UTRs, while most members of Cluster VIII contained long 3′ UTRs, which might be related with their mRNA stability, translation efficiency, and gene expressions [29,30,31]. In total, 10 different motifs were identified and shown in Figure 2, while motifs one, two, and three were the most conservative/common motifs of *EjSAURs*, as 76 *EjSAURs* contained these three motifs. The *EjSAUR* members assigned to the same clusters tend to have similar motifs, implying that they may play similar functions.

### 2.3. Cis Elements in the Promoters of EjSAURs

**Figure 3:**
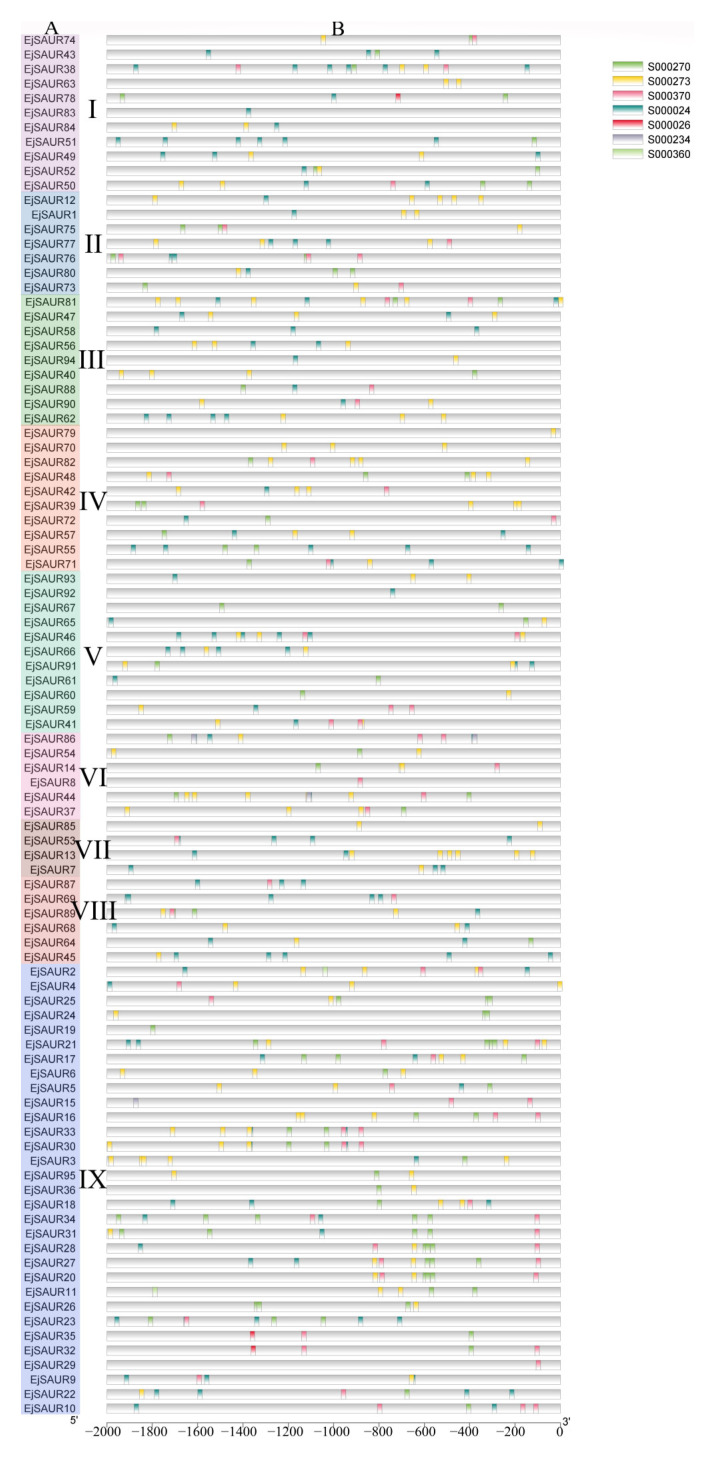
Distribution of major auxin-responsive *cis*-elements in the promoter regions of the *EjSAUR* genes. (**A**) *EjSAURs* of different clusters (I–IX). (**B**) Seven putative *cis*-elements are represented by different colors as indicated in the figure.

To elucidate the possible regulatory mechanisms under exogenous auxin stimulations, putative auxin-responsive *cis*-elements were searched in the 2000 bp promoter regions upstream of the transcription start site of the *EjSAUR* genes. In reference to a study in maize [3], the NEW PLACE website was used to search for seven *cis*-elements, including TGA-box (S000234), ARF binding (S000270), Dof protein binding (S000273), NDE element (S000360 and S000370), ASF-1 binding (S000024), and AuxRE (S000026). The results showed that all 95 *EjSAURs* contain at least one of the elements in their promoter regions (Figure 3). Except for AuxRE, TGA-box, and the NDE element (S000360), the remaining four *cis*-elements seemed universal in many *EjSAURs*. The presence of these identified *cis* elements suggests that these *EjSAURs* are potentially responsive to auxin stimuli and may play a role in plant hormone signaling.

### 2.4. Chromosomal Locations, Gene Duplication, and Synteny Analysis

**Figure 4:**
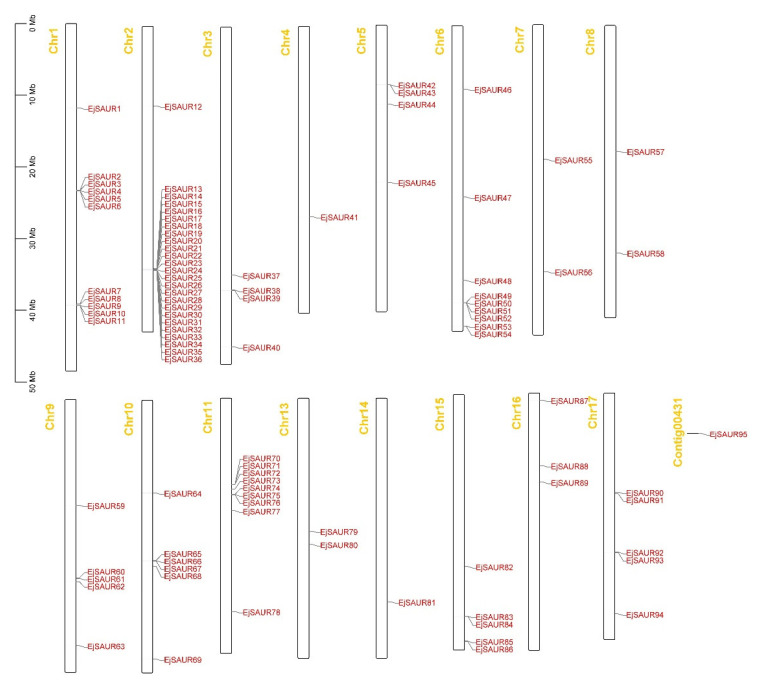
Gene locations of *EjSAURs*. The chromosome number is indicated at the top of each chromosome.

**Figure 5:**
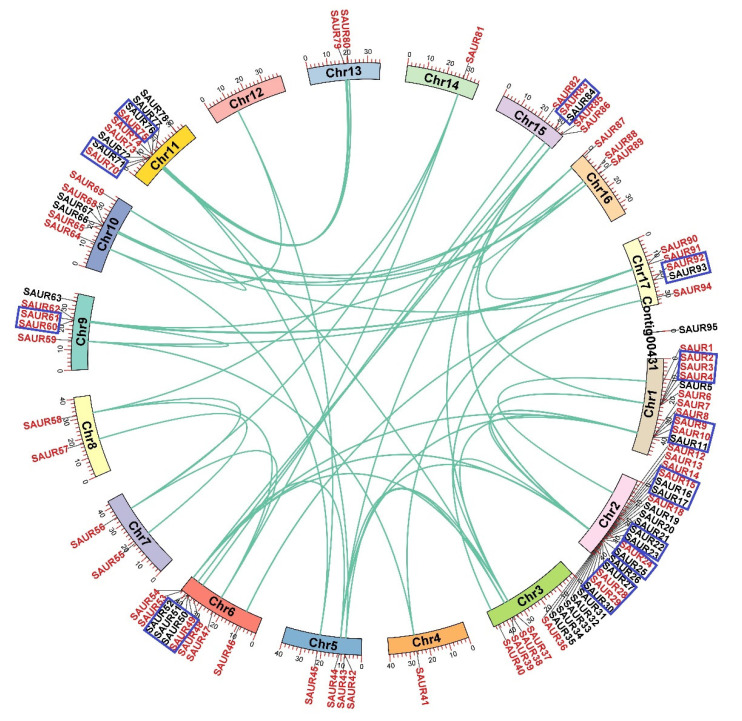
Tandem and segmental duplications of *SAURs* in loquats. The genes with a blue rectangule are tandem duplicated genes. The genes with a red font and connected with green lines are segmental duplicated genes.

The chromosomal locations of the 95 *EjSAURs* were plotted to a map (Figure 4). Obviously, they were non-evenly distributed throughout the 17 chromosomes. Surprisingly and interestingly, chromosome two contained a large cluster of *EjSAURs* (24 members). In contrast, some chromosomes contained only one *EjSAUR*, such as chromosomes four and fourteen. All tandem and segmental duplicated *EjSAURs* were plotted to a circos map (Figure 5). Further analysis revealed that between one and four tandem=duplication events of *EjSAURs* were observed on chromosomes one, two, six, nine, eleven, fifteen, and seventeen (Figure 5). As expected, chromosome two contained four tandem-duplication events, which may explain the large number of *EjSAURs* on this chromosome. In addition, a total of 61 segmental duplication pairs were identified by MCScanX [32], which may be related to the whole-genome duplication events during the evolution. Therefore, both tandem duplication and segmental duplication contributed to the expansion of *EjSAURs* in loquats.

**Figure 6:**
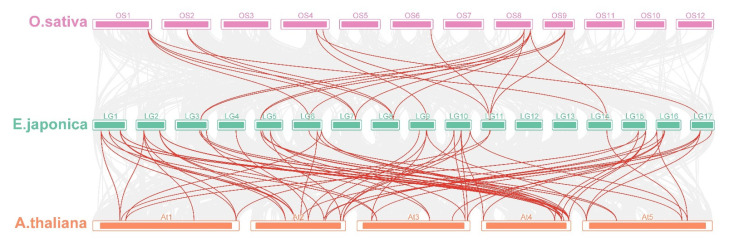
Multicollinearity analysis of *SAURs* from loquats, *Arabidopsis*, and rice. The red lines connect *SAUR* genes with a collinearity relationship. The grey lines connect other genes with a collinearity relationship.

The number of nonsynonymous substitutions per nonsynonymous site (Ka), the number of synonymous substitutions per synonymous site (Ks), and the Ka/Ks values were calculated for all duplicated gene pairs of *EjSAURs* (Table S2). The results show that almost all pairs had Ka/Ks < 1, implying that these genes were under purifying selection. However, the Ka/Ks values of the segmentally duplicated *EjSAUR49*/*EjSAUR83* (Ka/Ks = 1.30) and tandemly duplicated *EjSAUR49*/*EjSAUR50* (Ka/Ks = 1.53) were >1, suggesting that they were under positive selection and their functions may be differentiated or even that new functions could be evolved. By visualizing the *SAURs* with syntenic relationships between species, we found that the number of collinear pairs between loquats and *Arabidopsis* was much more than that between loquats and rice (Figure 6). A total of 45 *EjSAURs* had a syntenic relationship with the *SAURs* from *Arabidopsis* (85 collinear pairs), whereas only 12 *EjSAURs* had a syntenic relationship with the *SAURs* from rice (22 collinear pairs). There were nine *EjSAURs* (*EjSAUR38*/*39*/*42*/*43*/*47*/*62*/*74*/*81*/*90*) with collinear pairs in both *Arabidopsis* and rice, implying that they may share the same ancestral genes and have similar functions (Figure 6 and Table S2).

### 2.5. Expression Profiles of EjSAURs in Different Tissues

**Figure 7:**
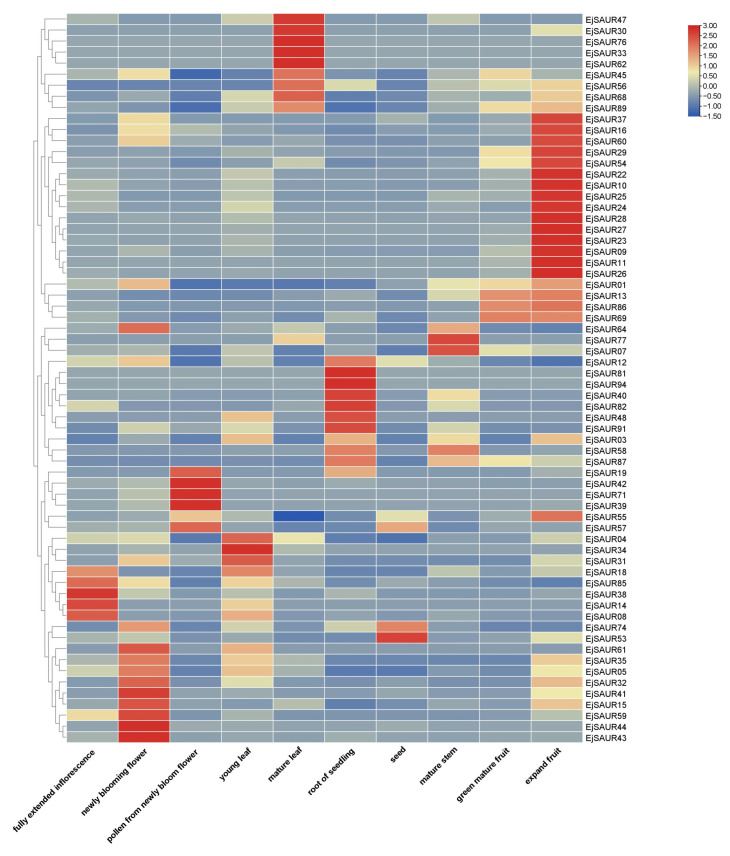
Tissue-specific expressions of *SAUR* genes in loquats. The transcripts per million (TPM) values were used for heatmap construction using TBtools.

To obtain a global view on the expression patterns of the *EjSAURs* in various organs and tissues, we re-analyzed a total of 10 transcriptomes of the inflorescence, flower, pollen, young leaf, mature leaf, root, seed, stem, expanding fruit, and green-mature fruit of a cultivar ‘Jiefangzhong’. An expression heatmap was constructed (Figure 7). The results show that many *EjSAURs* maintain high expression levels in the flower, mature leaf, root, and expanding fruit, implying that these *EjSAURs* play an important role in the growth and development of loquats, including fruit development. Interestingly, there were 14 *EjSAURs* (mostly from Cluster IX), such as *EjSAUR22*, *EjSAUR26*, and *EjSAUR29*, with very high expression levels only in expanding fruits, suggesting a vital role in fruit development. In addition, a minor proportion of *EjSAURs* were not expressed at all in any of the tissues, implying that they may not play important roles in the growth and development of loquats.

### 2.6. Observation of Fruit Development and Expression Patterns of Three EjSAURs

**Figure 8:**
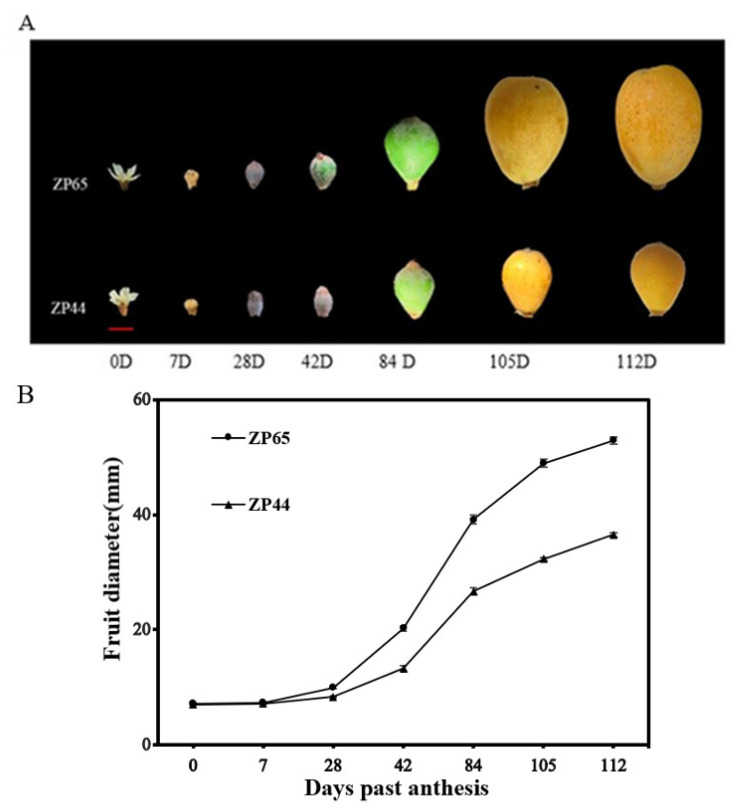
The fruit growth patterns of ZP44 and ZP65. (**A**) The receptacles and fruits at seven developmental stages. (**B**) Changes of fruit diameters along the seven developmental stages. 15 fruits at each stage for each line were measured. The error bars indicate standard errors.

The availability of two sister lines (ZP44 and ZP65) with contrasting fruit size performances enabled us to perform a detailed study of their fruit growth changes and evaluate the expression patterns of three *EjSAURs* (*EjSAUR22*, *EjSAUR26*, and *EjSAUR29*) across these fruit developmental stages, which were randomly selected out of the fourteen *EjSAURs* displaying high expression levels at the fruit-expanding stage. In total, seven stages were selected for fruit growth observations, including 0 day past anthesis (DPA), 7 DPA, 28 DPA, 42 DPA, 84 DPA, 105 DPA, and 112 DPA (Figure 8A). The results showed that the fruit size followed an ‘S’ curve pattern (Figure 8B). At earlier stages, the fruits grew relatively slowly, and no big difference was observed between the fruit sizes of ZP44 and ZP65. At 28-84 DPA, the fruits of both ZP44 and ZP65 expanded quickly, but ZP65 expanded at a much higher rate compared with that of ZP44. At around 105 DPA, the fruit sizes of both ZP44 and ZP65 started to plateau.

**Figure 9:**
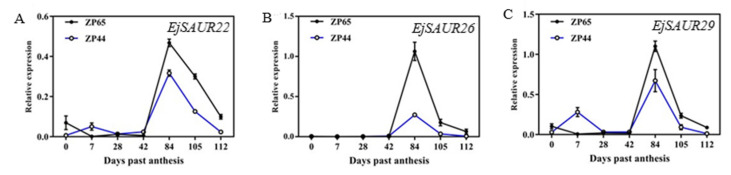
The expression patterns of three *EjSAURs* at different fruit developmental stages. (**A**–**C**) The expression patterns of *EjSAUR22* (**A**), *EjSAUR26* (**B**), and *EjSAUR29* (**C**). The error bars indicate standard errors.

The expression patterns of *EjSAUR22*, *EjSAUR26*, and *EjSAUR29* were investigated across the above seven developmental stages in ZP44 and ZP65. The three genes showed similar expression patterns in both ZP44 and ZP65: they maintained low expression levels at 0–42 DPA, started increasing after 42 DPA, reached to a peak at 84 DPA, and decreased thereafter (Figure 9A–C). However, the expressions of these three *EjSAURs* were all considerably higher in ZP65 (large-fruited) than that in ZP44 (small-fruited).

### 2.7. EjSAUR22, EjSAUR26, and EjSAUR29 Responses to IAA treatment

**Figure 10:**
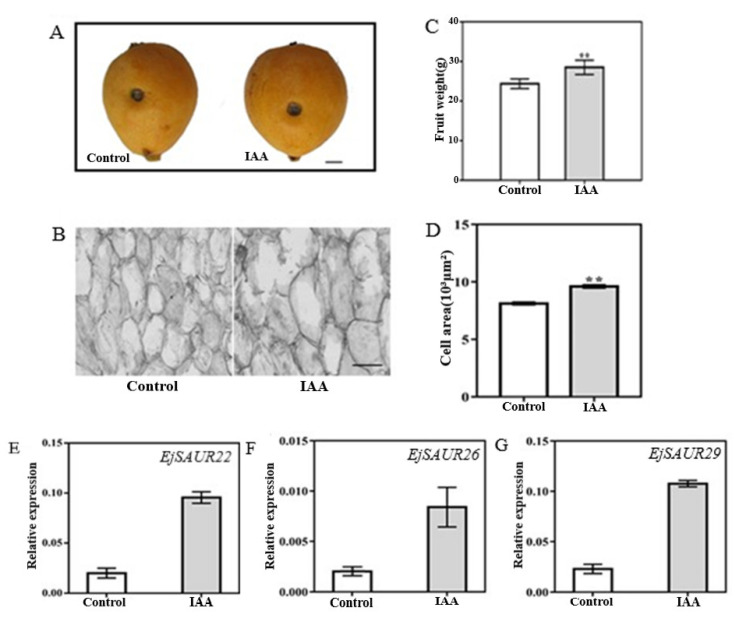
The influence of the exogenous IAA treatment on fruit growth and the expressions of three *EjSAURs*. (**A**) Photos of mature fruits from the control and IAA-treatment groups. The wounds indicate the positions where IAA was injected into. The bar represents 1 cm. (**B**) Microscopic observation of cell size in fruits from the control and IAA-treatment group. The bar represents 100 μm. (**C**) Comparison of fruit weights. More than 15 fruits were measured. ‘**’ indicates *p*-value < 0.01. (**D**) Comparison of cell areas. ‘**’ indicates *p*-value < 0.01. (**E**) The expressions of *EjSAUR22*, *EjSAUR26*, and *EjSAUR29*. The error bars indicate standard errors.

To investigate whether the above three *EjSAURs* were responsive to IAA, we injected IAA solution (10^−7^ M) into the fruits of the cultivar ‘Zaozhong No. 6′ at 63 DPA [23]. An exogenous injection of IAA into the fruits at this early expanding stage of ‘Zaozhong No. 6′ showed that the fruits (after reaching maturity at 116 DPA) were significantly larger (*p* < 0.05) than those of the control, and similar results were observed for fruit weight and cell area (Figure 10A–D). Importantly, at 14 days after treatment (DAT), the expressions of *EjSAUR22*, *EjSAUR26*, and *EjSAUR29* were all up-regulated in the IAA-treatment group compared with those in the control group, suggesting that they were all auxin-responsive (Figure 10E–G).

### 2.8. VIGS Support EjSAUR22′s Role in Cell Expansion and Fruit Size

**Figure 11:**
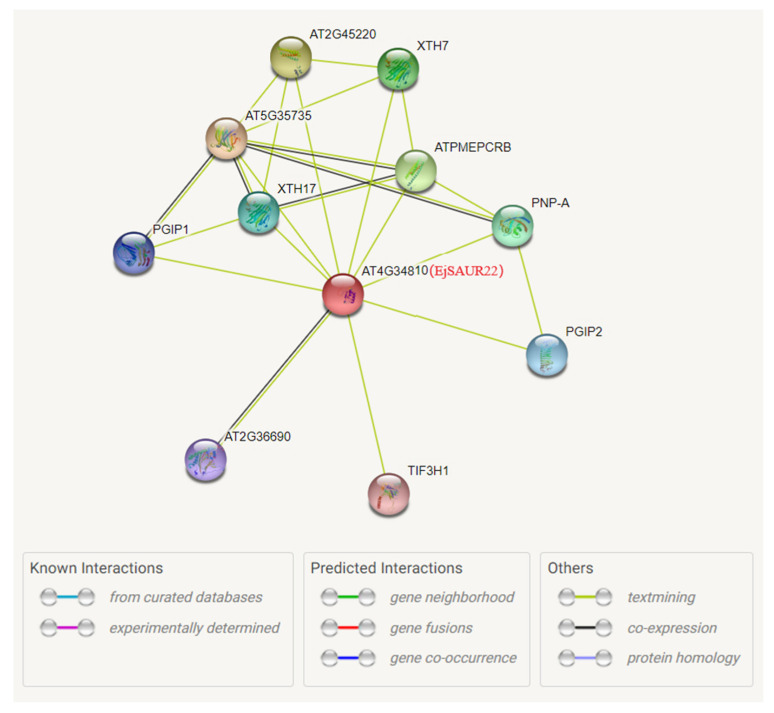
The predicted protein–protein interaction network of AT4G34810. The AT4G34810 collinear with *EjSAUR22* was used to investigate the potential interacted proteins using the STRING tool.

Among the above three *EjSAURs*, *EjSAUR22* showed a syntenic relationship with the gene AT4G34810 in *Arabidopsis*. Therefore, we further investigated its potential functions by analyzing the protein–protein interaction network of its collinear gene (AT4G34810) in *Arabidopsis* using the STRING database (Figure 11). Among the top interacting proteins with AT4G34810 were AT5G35735, an auxin-responsive family protein that may act as a catecholamine-responsive trans-membrane electron transporter, and AT4G02330, which may act in the modification of cell walls via demethylesterification of cell wall pectin. This analysis suggested that *EjSAUR22* may play a role in fruit expansion by regulating cell size. Subsequently, we further investigated the function of *EjSAUR22*.

**Figure 12:**
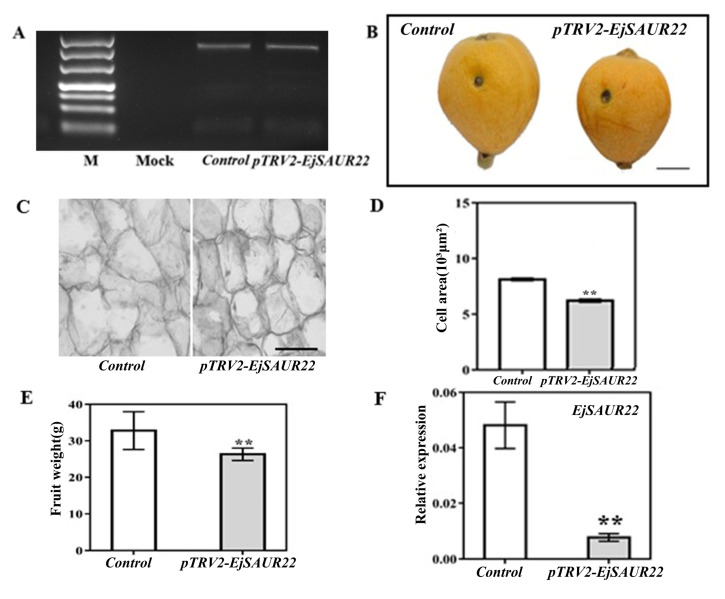
The influence of VIGS on fruit growth and expressions of *EjSAUR22*. (**A**) The detection of the coat-protein-coding sequence (cDNA) of TRV2. (**B**) Photos of mature fruits from the control and TRV2-EjSAUR22 groups. The bar represents 1 cm. (**C**) Microscopic observation of cell sizes in fruits from the control and TRV2-EjSAUR22 group. The bar represents 100 μm. (**D**) Comparison of cell areas. A total of 15 samples were used. (**E**) Comparison of fruit weights. More than 15 fruits were measured. (**F**) The expressions of *EjSAUR22*. The error bars indicate standard errors. ‘**’ indicates *p*-value < 0.01.

For validation of the function of *EjSAUR22*, virus induced gene silencing (VIGS) was performed at the early fruit-expanding stage (63 DPA) in fruits of ‘Zaozhong No. 6′ (Figure 12). The amplification of the coat-protein-coding sequence of TRV2 confirmed its presence in both TRV1+TRV2-empty (control) and TRV1+TRV2-EjSAUR (treatment) fruits, while there was no amplification in the mock fruits (no vector injection) (Figure 12A). After reaching maturity (116 DPA), fruits from the treatment group were found to be significantly smaller than those from the control group (Figure 12B,C, *p* < 0.01). Further histological observations revealed that the cells in the fruits of the treatment group were smaller than those in the control group (Figure 12D). As expected, at 14 DAT, the expression of *EjSAUR22* was significantly reduced (*p* < 0.05) in the treatment group compared with that in the control group.

Collectively, our results revealed that *EjSAUR22*, *EjSAUR26*, and *EjSAUR29* were responsive to IAA treatment, and *EjSAUR22* may play an important role in the fruit development of loquats by influencing the cell size and facilitating fruit expansion.

## 3. Discussion

Fruit quality traits are directly associated with consumer satisfaction and economic returns of fruit trees. Therefore, they have been gaining popularity in the research area. Fruit size is one of the important traits that influence the first impressions from consumers. The *SAUR* gene family regulated by auxin has proven to play a vital role in fruit development of many plant species [14]. However, this gene family has not been studied in-depth in loquats. Moreover, whether it is associated with fruit size or what members regulate fruit size remain understudied. In this study, we carried out a comprehensive analysis of the *SAUR* gene family in loquats. By assessing their transcriptional profiles in different tissues and at different fruit developmental stages comparing two sister lines with contrasting fruit sizes, a candidate *EjSAUR* gene was selected and further investigated. A combination of the IAA treatment experiment and functional validation using VIGS proved its role in regulating cell size and fruit expansion. These results not only provide genomic and genetic resources for fruit size breeding in loquats, but also lay a foundation for understanding the molecular mechanisms of auxin signaling and fruit expansion in loquats.

Previously, many other studies on the *SAUR* gene family revealed between 60–140 members in each plant species [14]. Similarly in the current study, a total of 95 *EjSAURs* were identified in the reference genome of ‘Seventh Star’. This number is relatively larger than that reported in *Arabidopsis* (72) [4], rice [9], and maize (79) [3]. It suggests that the *EjSAUR* family in loquats has experienced expansion during the evolutionary history. This is supported by the tandem duplication and segmental duplication events identified in the current study, which may be associated with the whole genome duplication in loquats [24]. Interestingly, chromosome two contained the largest number of *EjSAURs* compared with other chromosomes in loquats and harbored multiple tandem-duplication events, which may imply the special role of chromosome two in the growth and development of loquats. In accordance with the cases in many plants such as *Arabidopsis*, rice, and apples, the majority of the *EjSAURs* contain no introns. This seems to be a common feature of the *SAUR* gene family [25]. In accordance with studies in rice [9] and poplar [33], the *EjSAURs* showed tissue- or organ-specific gene expressions, indicating that their functions have probably diverged. The *EjSAURs* from this study were classified into nine clusters. In loquat, most *EjSAURs* in Cluster IX showed high expressions in expanding fruits, suggesting their role in fruit development. On the basis of collinearity analysis (Figure 6), we found that loquat shared more ortholog pairs of *SAUR* genes with *Arabidopsis* (85 pairs) compared with rice (22 pairs), which implied that the two dicots may share some duplication events. Among these collinear pairs, the protein–protein interaction network of the *Arabidopsis* gene AT4G34810 collinear with *EjSAUR22* was used to predict the potential function of *EjSAUR22*. Due to a potential role of *EjSAUR22* in regulating cell size based on this prediction, we selected it for VIGS assay, which supported its function in cell expansion and fruit expansion. Although it would be ideal to obtain transgenic loquat plants to investigate the functions of the *EjSAURs*, the genetic transformation system is still not well established in loquats. Furthermore, the long juvenile phase would also make this time-consuming.

Currently, fruit size breeding in loquats is still in its infancy, especially compared with several major fruit trees, such as apple and peach. The first reference genome of loquats was released in 2020 [27]. Therefore, the research at molecular and genomics level has just been initiated. In comparison, numerous approaches have already been widely applied in fruit size breeding in apples and peachs, including quantitative trait locus (QTL) mapping, marker-assisted breeding, genome-wide association study (GWAS), and genomic selection [34,35,36]. We believe the future direction of fruit size breeding in loquats will be integrating these advanced techniques with multi-omics, including genomics, transcriptomics, metabolomics, and proteomics, as well as with advanced phenotyping methods.

## 4. Materials and Methods

### 4.1. Plant Materials

All loquat trees (13-year-old) were grown in the *Eriobotrya* Germplasm Resource Preservation Garden (South China Agricultural University, Guangzhou, China) under regular management conditions. Two sister lines (ZP44 and ZP65) with contrasting fruit size performances were used for growth observations and gene expression assays at 0 days past anthesis (DPA), 7 DPA, 28 DPA, 42 DPA, 84 DPA, 105 DPA, and 112 DPA. For each analysis, 15 fruits at each stage for each line were used. Tissues close to the ovule or along the equatorial plane of the fruits were used as materials.

The cultivar ‘Zaozhong No. 6′ was used for IAA treatment. A total of 30 inflorescences at similar developmental stages and with similar sizes were selected. IAA was dissolved in 0.1% ethanol to obtain an IAA solution (10^−7^ M) for treatment, while the control used 0.1% ethanol solution. At the early fruit-expanding stage (63 DPA) in reference to our previous report [23], an Injex-30 injector (Thesera, Daegu, Korea) was used for injecting the solutions into the fruits along the equatorial plane. After reaching maturity at 116 DPA, fruits were collected for weight/size measurement and histological analyses. The fruit flesh tissues close to the ovule and along the equatorial plane were used. At 14 days after treatment (DAT), gene expression assays were performed.

### 4.2. Identification of the SAUR Gene Family

Among the three available reference genomes of *E. japonica* [24,27,28], the ‘Seventh Star’ genome [27] with the highest BUSCO score (99.1%, embryophyta_odb10) of the annotated gene models was used for genome-wide mining of *SAUR* genes. All SAUR proteins from *Arabidopsis* (AtSAURs) were searched and downloaded from the TAIR database (https://www.arabidopsis.org/ (accessed on 6 April 2021)). Two strategies were applied for identifying the *SAUR* genes in loquats. Firstly, the hidden Markov model (HMM) of the Auxin-inducible domain (PF02519) was downloaded from the Pfam database (https://www.ebi.ac.uk/interpro/ (accessed on 6 April 2021)) and used as a query to search against the protein sequences of the ‘Seventh Star’ with HMMER v3.3.1 from Sean R. Eddy and the HMMER development team (Cambridge, MA, USA) [37] under an e-value cutoff of 1 × 10^−5^. Secondly, the protein sequences of AtSAURs were used as the query to compare against the loquat proteins with BLASTP under an e-value cutoff 1 × 10^−5^ and identity >40%. Subsequently, the protein sequences of the above-identified genes were subjected to a further analysis using the Pfam database, the NCBI Conserved Domain tool, and the SMART database (https://smart.embl.de/ (accessed on 12 April 2021)) to confirm the presence of the SAUR domain. The ProtParam tool (https://web.expasy.org/protparam/ (accessed on 12 April 2021)) was applied to predict the physicochemical parameters, such as length, molecular weight, and isoelectric point of the identified SAUR proteins. The CELLO v2.5 server, developed by Chin-Sheng Yu and the team (Hsinchu, China), was used to investigate the subcellular localization [38].

### 4.3. Phylogenetic Analysis

A phylogenetic analysis was performed using the *SAUR* genes from *Arabidopsis*, rice, and loquats. The SAUR proteins from rice (OsSAURs) were obtained from a previous study [39]. The protein sequences were aligned using MAFFT [40]. MEGA11 was used for phylogenetic tree construction with the neighbor-joining (NJ) method and 1000 bootstraps, and other parameters were as default [41].

### 4.4. Chromosomal Locations, Gene Duplication, and Protein–-Protein Interaction Network

The physical locations of the identified *SAUR* genes on the 17 chromosomes of the loquat genome were visualized using TBtools [42]. Synteny analysis was performed using MCScanX [32] in TBtools for the *SAUR* genes between *Arabidopsis* & loquats and rice & loquats, as well as within loquats. Multi-collinearity analysis was performed and visualized using TBtools based on the results of the synteny analysis. Gene tandem-duplication events and segmental duplication events were also catalogued from the output of MCScanX. The nonsynonymous (Ka)/synonymous (Ks) analysis was performed using the Simple Ka/Ks Calculator within TBtools on the genes associated with tandem or segmental duplication events. The orthologs of *EjSAURs* from *Arabidopsis* were used to construct the protein–protein interaction networks with STRING (https://string-db.org/cgi/input.pl (accessed on 28 April 2021)).

### 4.5. Analysis of Gene Structure, Conserved Motifs, and cis-Elements of EjSAURs

The gene structure information was extracted from the GFF3 file of the ‘Seventh Star’ reference genome, which was visualized using TBtools. Conserved motifs were identified using MEME v5.4.1 (https://meme-suite.org/meme/ (accessed on 17 April 2021)). The 2 Kb sequences upstream of the *EjSAURs* were extracted and submitted to the New PLACE website (https://www.dna.affrc.go.jp/PLACE/?action=newplace (accessed on 17 April 2021)) for searching auxin-responsive *cis*-elements in their promoters.

### 4.6. RNA Extraction and qRT-PCR

RNA samples were extracted using the EASYspin Plus plant RNA extraction kit (Aidlab, Beijing, China). The first-strand cDNA was synthesized using the PrimeScript^TM^ RT reagent kit (TaKaRa, Kusatsu, Japan). qRT-PCR was performed following our previous report [43]. Primers for qRT-PCR were designed using BatchPrimer3 (v1.0) from Frank M You and the development team (Davis, CA, USA) [44]. All primer sequences are provided in Table S3. *EjRPL18* was used as the reference gene [45].

### 4.7. Virus Induced Gene Silencing

The coding sequences of *EjSAUR22* were cloned into the TRV2 vector to perform virus-induced gene silencing (VIGS) in the fruits of the cultivar ‘Zaozhong No. 6′. The VIGS experiment was carried out following the same method described previously [43], except that the *Agrobacterium tumefaciens* strain GV3101 was used. The TRV2-empty, TRV2-EjSAUR22, and TRV1 vectors were introduced into the *Agrobacterium tumefaciens* strain GV3101. At the early fruit-expanding stage (63 DPA), in reference to our previous report [23], an Injex-30 infector was used to inject the TRV1+TRV2:EjSAUR22 mixed *Agrobacterium* cells into the fruit near the equator. The TRV1+TRV2:Empty mixed *Agrobacterium* cells were used as a control. At 7 DAT, samples were collected to detect the presence and levels of the TRV2 virus. At 14 DAT, gene expression assays were performed.

### 4.8. RNA-Seq Analysis

To investigate the tissue-specific expressions of *SAURs*, our previously published transcriptome data from the cultivar ‘Jiefangzhong’ [24], covering tissues including the inflorescence, flower, pollen, young leaf, mature leaf, root, seed, stem, young fruit, expanding fruit, and mature fruit were re-analyzed using the ‘Seventh Star’ reference genome following the same method described previously [22]. The gene-expression heatmap was constructed using transcripts per million (TPM) values and TBtools.

## 5. Conclusions

Collectively, we performed a comprehensive analysis of the *EjSAUR* gene family in loquats, including investigating their physio-chemical features, evolutionary relationships, chromosomal locations, gene structures, *cis*-regulatory elements, and syntenic relationships. Through mining RNA-seq data, we obtained a group of *EjSAURs* highly expressed in expanding fruits that may play vital roles in the development loquat fruits. The results from the current study provide a theoretical foundation for future exploration of the features and functions of more *EjSAUR* genes. The *EjSAUR22* associated with cell expansion and fruit size could facilitate a deeper understanding of auxin signaling in the fruit development of loquats. Moreover, it may serve as a potential target for the manipulation of fruit size and accelerated breeding in loquats.

## Figures and Tables

**Table 1:** Summary of identified Small Auxin-up RNA (SAUR) gene family in loquat.

Gene Name	Gene ID	Location	Peptide Length	MW/Da	PI	Predicted Subcellular Location
*EjSAUR1*	EVM0023123	Chr1:11774882–11776212(−)	139	16,031.33	9.38	Nuclear(1.968)/Mitochondrial(1.116)
*EjSAUR2*	EVM0027975	Chr1:23253858–23254133(−)	91	10,303.12	9.37	Mitochondrial(2.134)
*EjSAUR3*	EVM0026311	Chr1:23277661–23278316(−)	102	11,240.81	6.39	Mitochondrial(1.514)/Nuclear(1.100)
*EjSAUR4*	EVM0036225	Chr1:23310700–23311301(−)	94	10,565.18	8.57	Extracellular(1.554)/PlasmaMembrane(1.101)
*EjSAUR5*	EVM0001621	Chr1:23313466–23314268(+)	93	10,399.74	6.96	Nuclear(2.403)
*EjSAUR6*	EVM0027340	Chr1:23320718–23321002(+)	94	10,519.81	6.89	Nuclear(2.747)
*EjSAUR7*	EVM0024197	Chr1:39105016–39105753(+)	151	16,938.55	9.03	Nuclear(2.201)
*EjSAUR8*	EVM0008023	Chr1:39155495–39156275(−)	105	12,020.02	8.51	Mitochondrial(2.036)/Cytoplasmic(1.594)
*EjSAUR9*	EVM0019503	Chr1:39309289–39309934(+)	101	11,396.06	6.56	Mitochondrial(1.637)/Nuclear(1.271)
*EjSAUR10*	EVM0040814	Chr1:39327746–39328520(+)	101	11,312.01	6.71	Mitochondrial(1.705)
*EjSAUR11*	EVM0039098	Chr1:39335634–39336324(+)	101	11,356.99	6.26	Mitochondrial(1.737)
*EjSAUR12*	EVM0025395	Chr2:11090245–11091063(−)	139	15,820.21	9.21	Nuclear(2.199)
*EjSAUR13*	EVM0010716	Chr2:33801351–33802064(+)	146	16,414	9.21	Nuclear(2.310)
*EjSAUR14*	EVM0039525	Chr2:33843069–33843505(−)	104	11,890.8	7.75	Mitochondrial(1.932)/Cytoplasmic(1.595)
*EjSAUR15*	EVM0005420	Chr2:33869889–33870586(+)	99	11,181.98	7.88	Mitochondrial(1.083)/Extracellular(1.060)
*EjSAUR16*	EVM0022268	Chr2:33874632–33875391(+)	100	11,280.98	6.06	PlasmaMembrane(1.800)
*EjSAUR17*	EVM0042792	Chr2:33884220–33884807(−)	127	14,144.29	6.71	PlasmaMembrane(1.550)/Extracellular(1.459)
*EjSAUR18*	EVM0004006	Chr2:33885766–33886265(+)	98	11,175.67	7.9	Mitochondrial(1.992)
*EjSAUR19*	EVM0002248	Chr2:33893889–33894164(−)	91	10,015.55	6.03	Extracellular(0.995)/Nuclear(0.962)/Chloroplast(0.957)
*EjSAUR20*	EVM0003298	Chr2:33906669–33906974(−)	101	11,214.91	5.23	Mitochondrial(1.203)/Extracellular(1.104)
*EjSAUR21*	EVM0028994	Chr2:33907908–33908180(+)	90	10,060.39	4.92	Cytoplasmic(1.643)/Nuclear(1.285)
*EjSAUR22*	EVM0027703	Chr2:33924785–33925382(+)	101	11,287.08	8.64	Mitochondrial(1.456)/Extracellular(1.106)/Nuclear(1.053)
*EjSAUR23*	EVM0022346	Chr2:33927004–33927649(−)	101	11,289.86	6.9	Mitochondrial(1.148)/Nuclear(1.073)
*EjSAUR24*	EVM0035336	Chr2:33934148–33934842(+)	91	9982.47	6.03	Nuclear(1.420)/Extracellular(1.227)
*EjSAUR25*	EVM0035171	Chr2:33936811–33937086(−)	91	10,019.61	8.73	Nuclear(1.371)
*EjSAUR26*	EVM0031995	Chr2:33938646–33939222(+)	101	11,267.96	6.82	PlasmaMembrane(1.157)/Mitochondrial(1.088)
*EjSAUR27*	EVM0031846	Chr2:33946504–33947087(+)	100	11,129.82	5.28	Mitochondrial(1.658)
*EjSAUR28*	EVM0002370	Chr2:33967678–33968177(+)	101	11,321.04	6.57	Mitochondrial(2.149)
*EjSAUR29*	EVM0015711	Chr2:33972636–33973301(+)	101	11,104.83	5.71	Extracellular(1.824)
*EjSAUR30*	EVM0038688	Chr2:34011539–34011841(+)	100	11,492.37	9.3	PlasmaMembrane(1.571)
*EjSAUR31*	EVM0025517	Chr2:34018506–34019087(+)	101	11,293.97	5.79	Mitochondrial(2.138)
*EjSAUR32*	EVM0044407	Chr2:34021147–34021686(+)	100	10,969.69	5.24	Mitochondrial(1.235)/Extracellular(1.134)
*EjSAUR33*	EVM0039690	Chr2:34032156–34032458(+)	100	11,512.44	9.46	PlasmaMembrane(1.862)
*EjSAUR34*	EVM0026210	Chr2:34039113–34039637(+)	101	11,302	6.72	Mitochondrial(2.080)
*EjSAUR35*	EVM0026900	Chr2:34041830–34042147(+)	105	11,625.53	7.77	Extracellular(1.488)/Mitochondrial(1.154)
*EjSAUR36*	EVM0001246	Chr2:34086922–34087275(+)	117	13,500.49	7.88	Nuclear(1.889)/Mitochondrial(1.787)
*EjSAUR37*	EVM0038271	Chr3:34602523–34602843(−)	106	12,197.03	9.3	Mitochondrial(2.380)
*EjSAUR38*	EVM0005442	Chr3:36683708–36684389(+)	120	13,571.39	5.33	Nuclear(1.986)
*EjSAUR39*	EVM0025157	Chr3:36746045–36748186(−)	183	20,384.85	6.24	Nuclear(2.636)
*EjSAUR40*	EVM0038485	Chr3:44606644–44607309(−)	128	14,229.45	6.13	Chloroplast(1.219)/Nuclear(1.118)
*EjSAUR41*	EVM0015619	Chr4:26550990–26551811(−)	142	16,013.1	5.66	Nuclear(2.491)
*EjSAUR42*	EVM0030373	Chr5:8343504–8346032(+)	183	20,272.91	6.38	Nuclear(2.709)
*EjSAUR43*	EVM0043234	Chr5:8361849–8362434(−)	121	13,671.57	5.11	Nuclear(1.523)/Mitochondrial(1.084)
*EjSAUR44*	EVM0017373	Chr5:11041105–11041507(+)	106	11,997.75	8.6	Mitochondrial(1.686)/Chloroplast(1.317)/Cytoplasmic(1.111)
*EjSAUR45*	EVM0039643	Chr5:21933735–21935000(−)	173	19,518.59	10.09	Mitochondrial(2.333)/Nuclear(1.759)
*EjSAUR46*	EVM0043534	Chr6:8833467–8843020(+)	144	16,158.07	9.08	PlasmaMembrane(2.867)
*EjSAUR47*	EVM0001296	Chr6:23893000–23895587(+)	170	19,202.14	9.32	Nuclear(1.616)/Extracellular(1.355)/Mitochondrial(1.262)
*EjSAUR48*	EVM0040221	Chr6:35531114–35531632(−)	172	19,075.91	8.83	Nuclear(2.561)
*EjSAUR49*	EVM0018324	Chr6:38647524–38647835(+)	103	11,754.81	9.76	Nuclear(1.809)/Mitochondrial(1.334)
*EjSAUR50*	EVM0003367	Chr6:38650612–38650929(+)	105	12,021.13	10	Mitochondrial(2.579)
*EjSAUR51*	EVM0009009	Chr6:38652604–38652915(+)	103	11,852.93	9.85	Mitochondrial(2.766)
*EjSAUR52*	EVM0026005	Chr6:38661854–38662329(+)	103	11,766.62	9.83	Mitochondrial(2.337)
*EjSAUR53*	EVM0012665	Chr6:41908807–41909985(+)	150	16,730.32	9.54	Mitochondrial(1.295)/Extracellular(1.238)
*EjSAUR54*	EVM0029547	Chr6:41946158–41947012(−)	105	11,990.81	6.9	Mitochondrial(1.711)/Cytoplasmic(1.321)
*EjSAUR55*	EVM0017455	Chr7:18784282–18785389(−)	226	25,481.7	10.14	Mitochondrial(1.378)/PlasmaMembrane(1.198)
*EjSAUR56*	EVM0025877	Chr7:34435942–34437008(+)	155	17,389.32	9.39	Nuclear(1.716)/Mitochondrial(1.423)
*EjSAUR57*	EVM0008209	Chr8:17546313–17546996(-)	169	19,146.49	9.8	Mitochondrial(1.960)
*EjSAUR58*	EVM0002293	Chr8:31723362–31723838(+)	153	17,274.22	9.56	Mitochondrial(2.006)
*EjSAUR59*	EVM0005588	Chr9:14721550–14722184(+)	152	17,317.65	7.65	Chloroplast(1.322)/Nuclear(1.146)
*EjSAUR60*	EVM0034387	Chr9:24812318–24812749(+)	133	15,062.18	7.84	Nuclear(2.062)
*EjSAUR61*	EVM0032278	Chr9:24905574–24906094(+)	166	19,047.25	9.57	Nuclear(2.880)
*EjSAUR62*	EVM0042993	Chr9:25377558–25377971(+)	137	15,658.85	6.07	Extracellular(1.704)/Nuclear(1.638)
*EjSAUR63*	EVM0021105	Chr9:34270371–34270790(−)	139	16,677.25	5.85	Mitochondrial(1.644)/Extracellular(1.343)
*EjSAUR64*	EVM0027800	Chr10:12919311–12920724(+)	171	19,170.14	10.1	Mitochondrial(2.625)
*EjSAUR65*	EVM0001701	Chr10:22309553–22310006(−)	138	15,798.33	7.71	PlasmaMembrane(1.540)/Nuclear(1.285)/Extracellular(1.056)
*EjSAUR66*	EVM0028728	Chr10:22335128–22335606(−)	135	15,510.61	9.63	PlasmaMembrane(2.371)
*EjSAUR67*	EVM0008977	Chr10:22405749–22406198(+)	106	12,495.67	8.55	PlasmaMembrane(1.849)
*EjSAUR68*	EVM0017929	Chr10:23141987–23142813(+)	124	14,583.63	8.54	Nuclear(1.893)
*EjSAUR69*	EVM0006441	Chr10:36037617–36038018(+)	133	15,649.71	6.67	Nuclear(2.017)
*EjSAUR70*	EVM0015952	Chr11:12020642–12021202(+)	186	21,109.31	9.1	Nuclear(2.785)
*EjSAUR71*	EVM0038556	Chr11:12028926–12029372(−)	148	16,773.47	8.55	Mitochondrial(1.690)
*EjSAUR72*	EVM0003137	Chr11:12032052–12032480(+)	142	16,444.39	9.24	PlasmaMembrane(1.521)/Mitochondrial(1.278)
*EjSAUR73*	EVM0023832	Chr11:12666841–12667188(+)	115	13,221.58	9.63	Mitochondrial(2.517)
*EjSAUR74*	EVM0000097	Chr11:13415260–13415932(+)	119	13,144.02	6.06	Mitochondrial(1.451)/Chloroplast(1.368)/Nuclear(1.136)
*EjSAUR75*	EVM0037519	Chr11:13443972–13444438(−)	118	13,507.83	9.45	Mitochondrial(2.147)
*EjSAUR76*	EVM0015267	Chr11:13450237–13450593(−)	118	13,512.04	9.52	Mitochondrial(2.192)
*EjSAUR77*	EVM0028342	Chr11:15696531–15696872(+)	113	13,066.42	9.4	Mitochondrial(2.054)
*EjSAUR78*	EVM0044617	Chr11:29707812–29708126(+)	104	12,044.19	9.52	Mitochondrial(1.827)/Nuclear(1.592)
*EjSAUR79*	EVM0017994	Chr13:18527573–18528148(+)	191	21,616.83	9.1	Nuclear(2.960)
*EjSAUR80*	EVM0008731	Chr13:20372332–20372661(−)	109	12,670.87	9.71	Mitochondrial(2.503)
*EjSAUR81*	EVM0036677	Chr14:28350218–28350736(+)	172	19,317.36	9.21	Extracellular(1.994)/Nuclear(1.716)
*EjSAUR82*	EVM0001339	Chr15:23948878–23949456(−)	173	19,287.14	9.23	Nuclear(2.277)
*EjSAUR83*	EVM0019162	Chr15:30879025–30879300(−)	91	10,594.44	9.62	Mitochondrial(1.726)/Nuclear(1.549)
*EjSAUR84*	EVM0006879	Chr15:30881712–30882023(−)	103	11,948.13	9.86	Mitochondrial(1.704)/Nuclear(1.409)
*EjSAUR85*	EVM0011555	Chr15:34348265–34349570(+)	150	16,912.54	9.69	Mitochondrial(2.123)
*EjSAUR86*	EVM0038736	Chr15:34388795–34389525(−)	105	12,107.98	7.83	Mitochondrial(1.870)/Nuclear(1.606)
*EjSAUR87*	EVM0006208	Chr16:993990–995003(−)	133	15,745.86	7.14	Nuclear(1.753)
*EjSAUR88*	EVM0027522	Chr16:10108766–10109161(−)	131	15,539.26	8.79	Mitochondrial(1.662)
*EjSAUR89*	EVM0021582	Chr16:12402979–12403859(−)	123	14,451.43	7.96	Nuclear(2.113)
*EjSAUR90*	EVM0039776	Chr17:13803945–13804328(−)	127	14,713.15	8.43	Extracellular(2.177)/Nuclear(1.739)
*EjSAUR91*	EVM0020457	Chr17:14042817–14043748(−)	149	16,909.35	9.25	Nuclear(1.794)
*EjSAUR92*	EVM0017269	Chr17:22150290–22150730(−)	146	16,326.86	9.24	PlasmaMembrane(1.510)/Nuclear(1.203)
*EjSAUR93*	EVM0039025	Chr17:22220726–22221196(−)	156	17,535.48	9.59	Nuclear(2.088)
*EjSAUR94*	EVM0010047	Chr17:30752223–30752606(−)	127	14,313.37	6.31	Nuclear(1.450)
*EjSAUR95*	EVM0035435	Contig00431:47729–48082(+)	117	13,500.49	7.88	Nuclear(1.889)/Mitochondrial(1.787)

## Data Availability

Not applicable.

## Supplementary Materials

The following supporting information can be downloaded at: https://www.mdpi.com/article/10.3390/ijms232113271/s1.
